# Study of Structural and Optical Properties of Electrodeposited Silicon Films on Graphite Substrates

**DOI:** 10.3390/nano12030363

**Published:** 2022-01-24

**Authors:** Muhammad Monirul Islam, Hajer Said, Ahmed Hichem Hamzaoui, Adel Mnif, Takeaki Sakurai, Naoki Fukata, Katsuhiro Akimoto

**Affiliations:** 1 Institute of Applied Physics, University of Tsukuba, Tsukuba 305-8573, Ibaraki, Japan; sakurai@bk.tsukuba.ac.jp (T.S.); FUKATA.Naoki@nims.go.jp (N.F.); akimoto.katsuhiro.gf@u.tsukuba.ac.jp (K.A.); 2 Alliance for Research on the Mediterranean and North Africa (ARENA), University of Tsukuba, Tsukuba 305-8573, Ibaraki, Japan; 3 Useful Materials Valorization Laboratory, National Centre for Research in Materials Science, Technological Park of Borj Cedria, B.P.73, Soliman 8027, Tunisia; hajer.said.doctorante@gmail.com (H.S.); mdmihi10@gmail.com (A.H.H.); mnifinrst@gmail.com (A.M.); 4 International Center for Materials Nanoarchitectonics, National Institute for Materials Science, 1-1 Namiki, Tsukuba 305-0044, Ibaraki, Japan

**Keywords:** SiO_2_, graphite substrate, Si nanowire, electrochemical reduction, microstructural properties

## Abstract

Silicon (Si) films were deposited on low-cost graphite substrates by the electrochemical reduction of silicon dioxide nanoparticles (nano-SiO_2_) in calcium chloride (CaCl_2_), melted at 855 °C. Cyclic voltammetry (CV) was used to analyze the electrochemical reduction mechanism of SiO_2_ to form Si deposits on the graphite substrate. X-ray diffraction (XRD) along with Raman and photoluminescence (PL) results show that the crystallinity of the electrodeposited Si-films was improved with an increase of the applied reduction potential during the electrochemical process. Scanning electron microscopy (SEM) reveals that the size, shape, and morphology of the Si-layers can be controlled from Si nanowires to the microcrystalline Si particles by controlling the reduction potentials. In addition, the morphology of the obtained Si-layers seems to be correlated with both the substrate materials and particle size of the feed materials. Thus, the difference in the electron transfer rate at substrate/nano-SiO_2_ interface due to different applied reduction potentials along with the dissolution rate of SiO_2_ particles during the electrochemical reduction process were found to be crucial in determining the microstructural properties of the Si-films.

## 1. Introduction

Silicon (Si), an indirect bandgap semiconductor in bulk form, is considered to play vital roles in a wide range of technologies (e.g., as electronic material in the field of electronics, and optoelectronics, and as an energy material in the field of photovoltaics, energy conversion, and energy storage devices) [1,2,3]. Particularly, solar cells, made of crystalline silicon are the prime contributor in meeting the market demand for renewable energy [4,5,6]. In addition, silicon-based anodes, due to their high theoretical capacity (4200 mAh/g), which is higher than current commercial graphite-based anodes, are considered promising anodes for lithium-ion batteries (LIBs) [7,8,9]. Nevertheless, challenges lie in the current commercial production of Si through the carbothermal reduction of silica (SiO_2_), which is associated with the high cost and environmental concerns [10].

Recently, several groups have been working on electrochemical methods, where Si is electrochemically deposited on conductive substrates through electrochemical reduction of SiO_2_ at high-temperature molten salts [11,12,13,14]. The method has been proven cost-effective and less energy-consuming compared to the current carbothermal method. Particularly, T. Nohira et al., [11] have reported contacting electrode method of electrochemical reduction, where a conducting metal is directly contacted to a SiO_2_ plate in CalCl_2_ melt at a high temperature of 850 °C. The electrochemical reduction of SiO_2_ starts through the transfer of electrons from contact metals to adjacent SiO_2_ at metal/CaCl_2_-electrolyte/SiO_2_ three-phase interface. In the report of T. Nohira et al., Molybdenum (Mo) wire was used as a conducting material directly contacted with SiO_2_. 

Electrodeposition of high purity Si film has been reported on silver (Ag) as conducting substrates, where SiO_2_ nanoparticles in high-temperature CaCl_2_ melt reduced on Ag-substrates through accepting electrons from Ag [15,16]. In addition, it has been found that the silver (Ag) substrates improve the crystallinity and photoactivity of the deposited Si films [15,17]. Nevertheless, Ag is a very expensive material to be used at large scale, and a less expensive substrate material would be preferable. The deposition of Si on low-cost substrates such as nickel, Mo, and tungsten (W) was also investigated [16,18,19]. However, most of these substrates were found unsuitable for Si electrodeposition. Indeed, they form silicides at operating temperature around 850 °C, which hinders the deposition of the pure Si layer. Recently, graphite appeared as a highly promising substrate-material for Si electrodeposition [20,21,22,23]. Besides its low-cost and availability, graphite has been considered relatively inert with silicon at 850 °C [24]. Moreover, graphite is more compatible with Si properties, and the combination of the benefits of Si and graphite was found to significantly improve the quality of the deposited Si on graphite. For instance, the ideal layered structure of graphite combined with the extremely high theoretical capacity of Si can effectively enhance the specific capacity, cycle stability, and rate capability of LIBs [25,26]. Therefore, deposition of Si films on graphite substrates is expected to be one of the most high-efficiency and low-cost production methods. Recently, direct electrolytic deposition of Si on graphite in molten CaCl_2_, and application of deposited Si for the fabrication of solar cells have been reported [20,21,22,23]. It was found that the morphology of the deposited Si-films can be controlled by the applied reduction potentials or the current densities during the electrochemical reduction of SiO_2_ feed materials [20,22].

Nevertheless, the quality and purity of the obtained Si-films have still remained issues to be improved, as they significantly affect the photo-response of the films and also the performance of the fabricated devices. In addition, microstructural properties including size, shape, and morphology of the electrodeposited Si-films are of great interest, since the application of Si in various technology is determined by its crystalline nature and the crystalline structure ranging from the nanowire and nanoparticle to the bulk crystals. For example, the quantum confinement effect in the nanocrystalline Si (both nanowire and nanoparticle) with increased bandgap and strong luminescent properties in the visible range make them attractive materials in the field of microelectronics as well as in energy conversion devices (such as solar cells [27] and secondary ion batteries [9]). On the other hand, single-crystalline bulk silicon has been extensively used in photovoltaics and other fields of electronics. Thus, for a successful application of the electrochemically obtained Si-films in practical devices, a detailed study of the Si-growth mechanism on graphite along with structural and morphological evolution of the Si films in relation to the growth parameters is of huge interest. H. Xie et al. [20] reported the evolution of morphology of Si-deposits on graphite from nanowires to microparticles in relation to the reduction potential, applied during electrochemical process. Peng et al. [22] reported that morphology of the Si-deposits on graphite can be controlled from nanowire to nanoparticles by adding Sn in the molten salt during the electrochemical process. The growth mechanism of granular-shaped Sn doped Si-film has also been reported. Nevertheless, the mechanism of the morphological evolution (e.g., nanowires to nanoparticles) of electrodeposited Si-films on graphite substrates is not well understood yet. Particularly, the formation mechanism of Si-nanowires on graphite has not been discussed. In addition, so far, there are no reports available on the optical properties, such as luminescent properties of the electrodeposited Si on graphite, which is worth investigating to enhance the material performance beyond the current level. Moreover, the quantum confinement effect in nanoscale electrodeposited Si on graphite has not been investigated yet.

In the current study, the crystallinity and structural and optical properties of the electrodeposited Si-films on graphite substrates were discussed in relation to the applied reduction potential during the electrochemical process. In addition, a general study about microstructural and morphological evolution of the electrodeposited Si-films, from the nanowire to the nanoparticle, has been performed in relation to the process parameters, such as electrochemical reduction potentials, substrate materials, and size of the SiO_2_ feed materials. Electrochemical kinetics during electrochemical reduction of SiO_2_ on graphite at high-temperature CaCl_2_ melt were also discussed. The quantum confinement effect in the electrodeposited Si-layer on graphite has been studied using laser power-dependent Si optical phonon mode in Raman spectra.

## 2. Materials and Methods

Electrochemical reduction of SiO_2_ has been performed in a three-electrode-based electrochemical cell, which has been designed to work at high temperatures. Detailed setup of the electrochemical system has been discussed previously [16]. In the three-electrode-based setup, a graphite-rod (99.995%, 3 mm dia., Sigma-Aldrich, Milwaukee, Wisconsin, USA) was used as a working electrode (WE or cathode) that also served as a substrate for the electrochemical experiment. As for the counter electrode (CE or anode), another graphite rod with a similar specification has been used, while a molybdenum (Mo) rod (99.95%, 1.5 mm dia., Nilaco, Tokyo, Japan) has been used as a reference electrode (RE). All of the three electrodes were set up in a small Al_2_O_3_ crucible, which was set inside a long quartz cell, covered with a stainless-steel cap, and equipped with necessary feedthroughs for electrodes, Ar gases, and thermocouples.

Deposition of Si on graphite-WE was obtained through electrochemical reduction of SiO_2_ in high-temperature molten salt. Commercial SiO_2_-nanoparticles (nano-SiO_2_) with diameters in the range of 5–15 nm of particle size, and purity of 99.5% (Sigma-Aldrich, Tokyo, Japan) were used as feed materials for the reduction. For the experiment, 515 mg of nano-SiO_2_ were taken in an Al_2_O_3_-crucible and mixed with 30 g of anhydrous CaCl_2_ electrolyte (molten salt) with a purity level of 97% (Sigma-Aldrich, Japan). Prior to the mixing, CaCl_2_ was dried at a temperature of 550 °C for 5 h under argon (Ar) gas flow to confirm the removal of any possible moisture. The flow of Ar was also maintained (flow rate, 150 mL/min) to confirm the inert atmosphere during the electrochemical reduction experiment of nano SiO_2_. The complete electrochemical setup was placed inside a furnace and the temperature was progressively raised to 855 °C. When CaCl_2_ melt at 855 °C, the temperature was held constant for the electrodeposition process. Electrochemical behaviors of nan-SiO_2_ were first examined by cyclic voltammetry (CV) at a scan rate of the potential set as 10 mV/s using an automatic polarization system (HSV-110, Hokuto Denko Co. Ltd., Tokyo, Japan). Then, a constant potential reduction (chronoamperometry, CA) was applied for two hours (2 h) using the same polarization system. After the electrodeposition experiment, deposited Si-films were ultrasonically washed with HCl solution (0.1 mol/L) for 5 min and then rinsed with pure water to remove CaCl_2_ melt and any unreduced nan-SiO_2_ if exists.

To investigate the effect of the reduction potential on the microstructural evolution of electrodeposited Si films on graphite WE, electrochemical reduction of nano SiO_2_ was performed under various reduction potentials. Crystallinity and morphological properties of the deposited Si- films were investigated using microscopic Raman (μ-Raman) spectroscopy, microscopic photoluminescence (μ-PL), scanning electron microscope (SEM), and X-ray diffraction (XRD) technique. A Nd:YAG laser line with a wavelength of 532 nm and having a focus size of several μm was used for the PL and Raman measurement. PL Spectra were analyzed by a grating monochromator and detected using a Si-CCD and a InGaAs detector to cover the wavelength of interest. During XRD analysis, 2θ angles were varied from 10°–90° by Philips X’pert system. The beam power was operated at 45 kV and 40 mA with Cu-Kα (α = 1.541837 Å) radiation. All of the characterization of Si-films have been carried out at room temperature (RT).

## 3. Results and Discussions

### 3.1. Cyclic Voltametery

**Figure 1:**
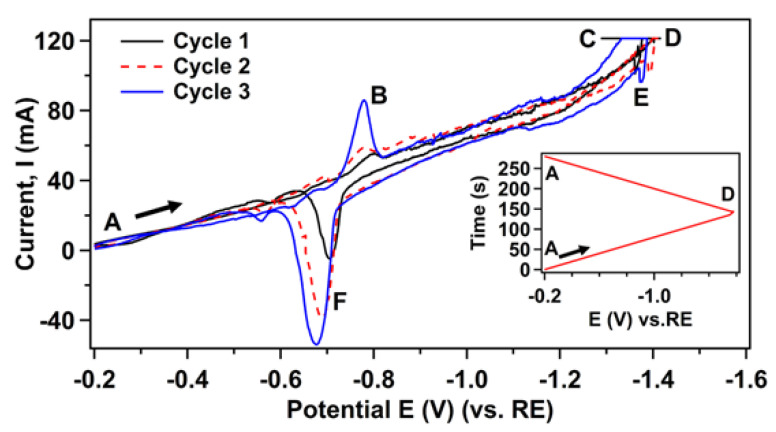
Cyclic voltammogram (CV) of the graphite electrode in the CaCl_2_-melt containing nano-SiO_2_ at 855 °C. Potential *E* was applied on the graphite electrode vs. Mo-RE at a scan rate of 10 mV/s. The saturation of the current above 120 mA at a higher potential region is due to exceeding the limit of the polarization system. The inset of the figure shows the first cycle of the applied negative potential with respect to the Mo-RE.

Initially, we have performed cyclic voltammetry (CV) to investigate the mechanism of the electrochemical process involving the reduction of nano-SiO_2_ to Si on the graphite -WE in CaCl_2_ melt at 855 °C. CV has been performed by a linear potential sweep (*E*), applied on the graphite-WE with respect to the Mo reference electrode (Mo-RE) at a scan rate of 10 mV/s. Shown in Figure 1 is the CV curve where the x-axis represents the applied potential (*E*), while the y-axis is the corresponding resulting current. The arrow in the CV curve indicates the direction in which the potential is scanned. The potential scan was repeated for three-cycles, and the inset of Figure 1 shows one complete cycle of the potential sweep. In one complete cycle, the potential is firstly scanned from the starting potential of −0.2 V (point A) to the more negative potential of −1.6 V (point D). This is referred to as the cathodic trace. The scan direction is then reversed, and the potential is swept positively back to (−0.2 V, point A), referred to as the anodic trace. As seen from the figure, during the cathodic scan (−0.2 V to −1.6 V) a gradual increase of the current appears starting after −1.0 V vs. graphite RE (cycle-1). We attribute this increase of the current due to the reduction of the nano-SiO_2_, where SiO_2_ starts to be reduced locally to make Si at the graphite WE.

Further scanning to more negative potential than −1.3 V leads to the faster increase of the reduction current up to the limit of the potentiostat used in this experiment. This high reduction current at this potential has been attributed to the reduction of CaCl_2_ to Ca^2+^ (cycle-1, point C) and the formation of Si-Ca alloys [19]. Indeed, according to the phase diagram of the Si–Ca system, several Si-Ca compounds occur at 855 °C [28]. When the sweeping potential is reached at point D, the scan direction is reversed, and the potential is scanned in the positive (anodic) direction. During the reversed scan, some features are observed around −1.4 to −1.5 V (point E), which could be attributed to the anodic dissolution of Si-Ca compounds. Thus, features at point C and E might be attributed to the formation–deformation of Si–Ca alloy [16]. As the applied potential becomes more positive, the Si present at the graphite- WE electrode is oxidized back to SiO_2_ making a thin layer of SiO_2_ on the graphite-WE. An anodic current peak is therefore observed (point F). After continuing to the cathodic scan (−0.2 V to −1.6 V) at cycle-2, a new reduction peak appears around −0.8 V vs. graphite RE (cycle-2, point B), which corresponds to the re-reduction of the formed thin SiO_2_ layer deposited during the previous cycle. A similar tendency of the appearing of the reduction and oxidation peaks was observed at the CV on Mo and Ag electrode in SiO_2_ containing the CaCl_2_ melt [15,16,18]. With a repetition of the scan cycles, both reduction and oxidation peaks shift to corresponding higher potential making larger peak separation. This roughly suggests that electrochemical kinetics becomes more slower with time in the next cycle. In the case of the cathodic scan, this slow electrode kinetics is associated with the requirement of the higher reduction potential, thus making shifting of the peaks to a more negative potential. In addition, as the cycle scan is repeated (cycle-2, cycle-3), the peak height of both reduction and oxidation peaks gradually increases. This suggests a broadening of the reduction-oxidation interface due to the formation of Si-based diffusion layer on graphite [29]. Once we confirmed the required potential for the reduction of nano SiO_2_, we performed an electrochemical reduction at a constant potential (i.e., the chronoamperograms (CA) technique for the formation of electrodeposited Si-layer on the graphite-WE). In a three-electrode based electrochemical system, CA is performed by applying a step potential (in fact, over potential which is more than the required value to initiate the reduction reaction) between WE and RE, and the resulting reduction current, which is proportional to the applied step potential, plays a role in the electrochemical reduction of SiO_2_ in molten CaCl_2_. To investigate the effect of the reduction potential on the quality and microstructural properties of the deposited Si-layers, we applied different constant reduction potentials for the electrodeposition of Si from nano-SiO_2_ on graphite-WE. The constant reduction potentials are conducted in the range of *E* = −1.15 to −1.25 V with respect to Mo-RE. Based on the voltammetric results, these potential values are well above the required potential (overpotential) for SiO_2_ reduction (i.e., above −1.00 V). However, below enough for the reduction of Ca^2+^ (−1.3 V), it was possible to avoid the formation of Si-Ca alloy in the deposited Si-films. In fact, electrochemical reduction with applied reduction potential, *E* = −1.00 V resulted in no formation of Si-layer on the graphite as confirmed visually and also with Raman measurement. Thus, in this paper, we define three reduction potentials, namely *low potential* (*E* = −1.15 V), *moderate potential* (*E* = −1.20 V), and *high potential* (*E* = −1.25 V). Material properties of electrodeposited Si-layer were investigated in relation to these defined reduction potentials.

### 3.2. Structural and Crystal Properties

**Figure 2:**
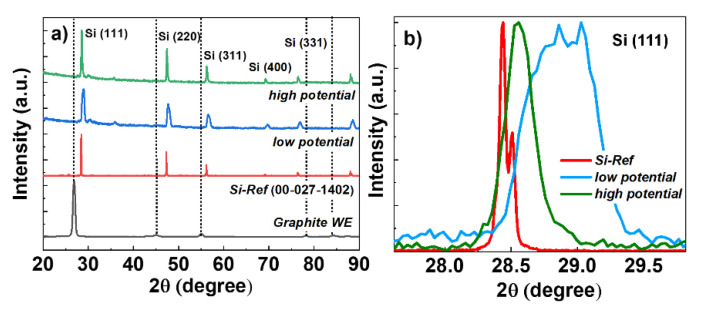
(**a**) XRD patterns, taken at θ–2θ mode, of the Si powders from the samples electrodeposited on graphite substrates with *low* and *high* reduction potentials vs. Mo-RE. The intensity of the XRD of the graphite rod (WE) has been reduced to a factor of 1/10. (**b**) Normalized XRD pattern of (111) peaks.

Shown in Figure 2a is the XRD plot of the samples prepared by constant reduction potential of *low potential* (*E* = −1.15 V) and *high potential* (*E* = −1.25 V) versus Mo-RE. To avoid the strong peaks from graphite substrates in the XRD patterns, we have peeled off the Si-layers from the graphite-WE and ground them to powder to conduct the XRD experiment. XRD of the graphite rod after peeling off the Si-layers was also taken. In addition, to confirm the peak positions of the electrodeposited Si, the XRD pattern of a crystalline Si has also been plotted in the same figure as a reference (ICDD standard: 00-027-1402). As seen from the figure, XRD patterns clearly confirm the formation of silicon (Si) phases on graphite substrates. In addition, it can be seen that with an increase of the reduction potential from *low potential* to *high potential*, the intensity of the XRD peaks increases and peaks become sharper with a decrease of the full width at half maximum (FWHM). Moreover, Gaussian analysis of the (111) diffraction plane (Figure 2b) shows a peak shift to 2θ = 28.88° for the sample deposited at *low potential* compared to the reference peak position at 2θ = 28.44°. Peak shift decreases to 2θ = 28.56° for the sample deposited at *high potential*. Roughly, this peak shift to the higher angle compared to the reference Si-peak can be attributed to the lattice contraction or formation of microstrain in the Si-deposits, the magnitude of which decreases with an increase of the reduction potential. The crystallite sizes of the Si-deposits are correlated with the broadening of the XRD peaks and can be calculated using Scherrer’s equation [30]:

(1) $$D=\frac{K\lambda }{\beta cos\theta }$$

Here, *D* is the mean size of the crystallites or coherently ordered crystalline domain (nm), *K* is a constant related to crystallite shape, normally taken as 0.94 for cubic structure, *λ* is the X-ray wavelength with the value of 1.54056 Å in the case of CuKα1 radiation, *β* is the full width at half the maximum (radians) of the X-ray diffraction peak, and *θ* is the Bragg diffraction angle (in degree). Gaussian fitting of the (111) peaks in both samples was used to determine FWHM and peak position. The calculated results of the crystalline size using the fitting parameters were found as 34.14 nm and 15.15 nm for Si samples, electrodeposited at *high potential* and *low potential*, respectively. It should be noted that Scherrer’s formula provides only the lower limit of the crystallite size, which may be smaller or equal to the grain size or particle size. In addition, the broadening of the XRD peaks might also be correlated to the inhomogeneous lattice strain as we mentioned above, and also to lattice defects. In fact, a rough estimation of the microstrain (ε) based on the (111) diffraction plane, and using the equation [31],

(2) $$\varepsilon =\frac{\beta }{4tan\theta }\;$$

gives a value of *ε* = 4.3 × 10^−3^ radian and 9.6 × 10^−3^ radian for the Si deposits obtained at *high potential* and *low potential*, respectively. Hence, it can be assumed that increased negative reduction potential leads to the increase of the particle size along with the increased crystallinity of the electrochemically obtained Si-deposits.

**Figure 3:**
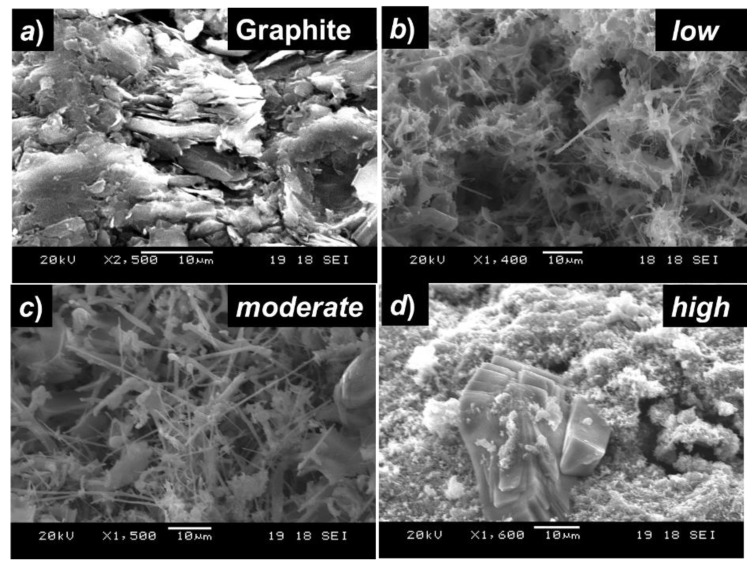
SEM image of the electrochemically deposited Si films on graphite substrates (**a**) surface of the graphite substrate before electrodeposition, (**b**) Si-films obtained with applying reduction potential at the graphite-substrate set as *low potential* vs. Mo-RE. (**c**) Si-films obtained at *moderate potential*, (**d**) Si-films obtained at *high potential*.

Later, we have investigated the microstructural properties and morphology of the electrodeposited Si-layers on the graphite-substrates using SEM as shown in Figure 3. As seen from Figure 3a, before deposition, graphite substrate shows a smooth morphology with a layered structure. Si-layer obtained with applying a *low potential* mainly consisted of Si nanowires [Figure 3b], with a diameter estimated from SEM image is up to hundreds of nm. With an increase of the reduction potential to *moderate potential*, the morphology of the deposit transfers to a mixture of Si nanowires of increased diameters and agglomerated Si nanoparticles, as can be seen from Figure 3c. However, with increased reduction potential to *high potential*, Si nanowires disappeared and relatively larger grains of Si, embedded into the matrix of Si-nanoparticles were observed [Figure 3d]. Thus, the size, shape, and morphology of the electrodeposited Si-layers on graphite substrates seem to be dependent on the applying reduction potential during electrochemical reduction of nano-SiO_2_. H. Xie et al. [20] also reported a similar dependence of the morphology of Si-films on the reduction potentials during electrochemical reduction of nano-SiO_2_ on graphite substrates, conducted in a two-electrode-based electrochemical system. The size and morphology of the deposited Si-nanowires on graphite also depend on the cathodic current densities, as has been reported by Peng et al. [22], where their group performed constant current electrolysis of SiO_2_ in molten KCl-KF-1 mol% K_2_SiF_6_ salt at 650 °C.

**Figure 4:**
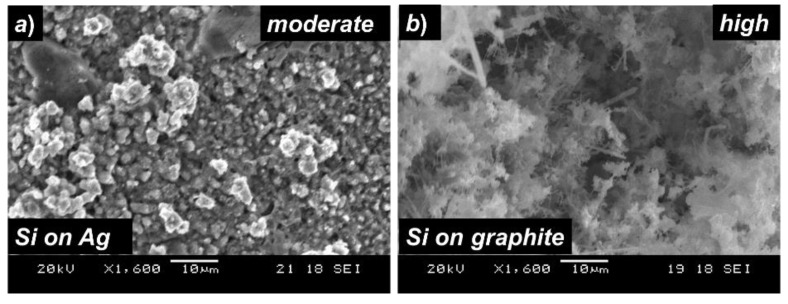
(**a**) SEM image of the electrochemically deposited Si films on a silver substrate obtained with applying reduction potential equivalent to *moderate potential* (**b**) SEM image of the electrochemically deposited Si films on graphite substrates from the reduction of larger-sized SiO_2_ particles, and with applying reduction potential equivalent to *high potential*.

The similar group also reported that during electrolysis, the addition of a small amount of tin (Sn) into the liquid melt assist in the formation of a dense and thick layer of Si on the graphite substrates, where morphology shows larger grains of Si, with no formation of Si nanowires. The author attributed the lateral growth (granular) of the Si particles to the mediator role of Sn, which is associated with enhanced surface diffusion properties at the growth temperature of 650 °C, mainly due to its metallic bonding compared to the only covalently bonded Si in the absence of Sn. In fact, in our previous study [16], we have investigated the effect of reduction potential on the electrochemical formation of Si-layer on Ag substrates from nano-SiO_2_. Although the crystalline size was observed to be increased from nanoscale to the microscale level with an increase of the applied potentials, no nanowire formation has been observed even at the lower reduction potential of *E* = −1.00 V (with respect to graphite-RE). In this study, to confirm the effect of substrate materials on the morphology, we have deposited Si-layer on an Ag-foil with a reduction potential equivalent to *moderate potential* (*E* = −1.20 V with respect to graphite-RE) from similar nano-SiO_2_ as feed materials, while keeping the other growth parameters similar to the conditions used in case of graphite substrates. Shown in Figure 4a is the SEM image of the electrodeposited Si-layer on the Ag-substrate. As seen from the figure, the morphology of the deposited Si-film shows the granular shapes with no formation of nanowires. In fact, electrochemical formation of the Si-layer on other metal-substrates (e.g., Mo, Ni) has been reported to show granular growth of deposited layers [18,32]. Thus, it can be considered that substrate-metals at a higher growth temperature decide the formation and growth orientation of the nuclei at the initial stage and play a role in determining the final shape and morphology of the grown films. The rate of nuclei formation, orientation, and size of the final grains should also then be related to the electron transfer rate from metal to SiO_2_, which in turn might be related to the mobility and diffusion properties of metal into SiO_2_ at a higher growth temperature. The formation of any liquid droplets or mixed phase of metal-Si at the growth temperature may also affect the morphology and grain size of the deposited films.

However, it is interesting to note that electrochemical reduction of solid silica (in any forms, e.g., quartz substrates, SiO_2_ pellet, etc.) with in-contact with metals including Mo, Ni, W etc. in high-temperature molten salt has been reported to form nanowires, especially at the region of solid-silica/metal-contact interface [11,19,33,34]. Thus, it can be assumed that the size of the crystalline domain of the feed materials may also play roles in defining the morphology of the Si-layers during the electrochemical reduction process. Thus, to understand the size-effect of the SiO_2_ particles on the morphology of the electrochemically obtained Si-layer on the same graphite substrate, we have deposited a Si-layer on the graphite-substrate using SiO_2_ feed material with the size of the particles in the range of 300–600 µm. A reduction potential of *E* = −1.25 V that is equivalent to *high potential* has been applied on graphite-WE with respect to Mo-RE, keeping other growth conditions similar to previous experiments with nano-SiO_2_. Shown in Figure 4b is the SEM surface image of the obtained Si-layer on graphite-substrate, electrochemically reduced from SiO_2_ of larger-sized particles. In comparing to the Si-layer obtained from nano-SiO_2_, the Si-layer deposited with larger SiO_2_ particles shows smaller grain size in nanoscale levels, obtained by applying a similar reduction potential. It has been reported that due to the insulator nature of SiO_2_, the electrochemical reduction process of SiO_2_ to Si at the graphite/SiO_2_ interface is kinetically slow even at a higher reduction temperature of 855 °C [11]. In fact, the gradual increase of the cathodic current in the CV diagram at Figure 1 also supports that the reaction kinetics are relatively slow at graphite-WE in the high-temperature melt of SiO_2_ and CaCl_2_. Thus, the faster dissolution rate of nano-SiO_2_ in contact with the graphite-WE at higher growth temperature is associated with faster reduction of SiO_2_ and formation of relatively larger grains at the given growth time. On the other hand, a relatively slower dissolution rate of SiO_2_ with the larger-sized particles is responsible for a slower reduction rate due to reduced capacity of receiving electrons from substrates, thus making the grains of the Si-films smaller in size. The above observation also explains the formation of small-sized Si-nanowires in solid silica in contact with metal, which could be attributed to the slow reduction rate of solid silica at a given potential. In addition, X. Yang et al. [23] studied the deposition of Si layers on the graphite substrates from nano-SiO_2_ in the mixture of CaCl_2_ (4.8 mol%)-CaO (3.9 mol%) molten salt at high temperature. The author reported the formation of dense and crystalline Si, mainly due to the high dissolution rate of nano-SiO_2_ originated from the formation of soluble Si^iv^-O complex anion with nano-SiO_2_ and O^2^^−^ in CaO. Morphology of the obtained Si was mainly granular in size without formation of nanowires. Thus, it can be assumed that an increased dissolution rate of nano SiO_2_ in the presence of CaO is mainly responsible for faster reduction rate and, thus, the formation of granular morphology, while nano-SiO_2_ in molten salt without the addition of CaO is associated with relatively slow reduction rate, and thus should yield the formation of nanowires.

**Figure 5:**
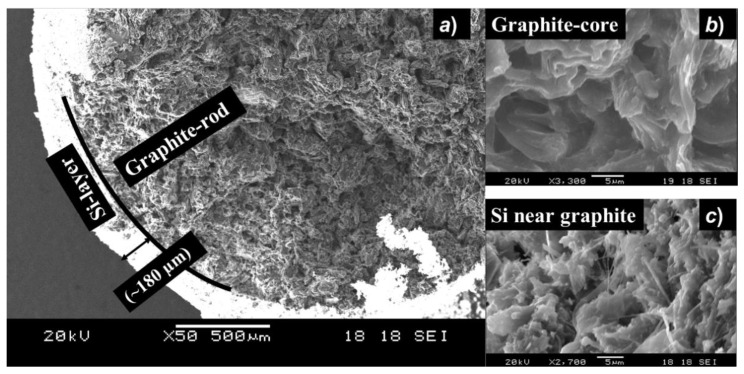
(**a**) Cross-sectional SEM image of the Si-coated graphite rod after electrochemical reduction of nano SiO_2_ at *low potential* and washed with HCl, (**b**) magnified image of the graphite core, (**c**) magnified image of the Si-layer close to the graphite-rod.

Finally, to understand the growth rate and thickness of the electrodeposited Si-layer, we have performed a cross-sectional SEM of the Si-coated graphite substrate. Figure 5 shows cross-sectional SEM images of the similar sample (grown with *low potential*), the surface of which has been shown in Figure 3b.

As seen from Figure 5a, the core consists of the graphite-rod (WE), showing part of a well-defined circle (diameter 3 mm) with a darker color. The core is covered with the deposited Si layer showing brighter color with 100–200 μm of thickness. In the case of graphite substrate, H. Xie et al. [20] reported a formation of a transition layer at the Si/graphite interface which is consisted of a mixture of elemental Si and C and also SiC phase. The formed SiC could help to the adhesion of deposited Si-layer on the substrate and help to grow thick films. However, it should be noted that the thickness of the Si-films around the graphite rod is not uniform [Figure S1]. A constant flow of Ar gas (150 mL/min) during the electrochemical reduction process might produce strong convection in the melt of SiO_2_ and CaCl_2_, mainly due to the temperature gradient at the melt surface and the bottom part of the electrochemical cell. This nonuniform convection affects the diffusion rate of SiO_2_ from different locations of the electrochemical cell towards the graphite-WE, and thus affects the reduction rate. Furthermore, the relative position of the graphite-WE and Mo-RE in the electrochemical cell may affect the reduction rate at different locations of the graphite rod, thus making films with non-uniform thickness. Nevertheless, although the thickness of the Si-layer is very large, the morphology of the obtained Si-layer around the graphite-rod (WE) may not be dense and shows a dendrite-type structure. In addition, Figure 5b shows a magnified SEM image of the core of the graphite-rod (WE), while Figure 5c shows a magnified SEM image of the Si-coating, roughly close to the graphite substrate. It is interesting to note that although the surface of the sample is composed of mainly Si nanowires with very small dimensions [Figure 3b], Si particles close to the graphite-rod seem relatively larger in size with very few portions of nanowire phases. It should be noted that the purity of the electrodeposited Si-films in this study has not been confirmed. However, as the kinetic reaction of Si and C is typically slower at a growth temperature of 855 °C, except for the interface at Si/graphite, the surface of the deposited Si-layer is assumed not to contain any mixed phase of Si and C as well as any elemental C diffused from the substrate. Nevertheless, the surface of the Si-deposits may contain a very thin amorphous type SiO_2_ phase, originated from the ambient air after HCl washing or through the reaction of evolved O^2−^ and newly-formed Si nuclei during the electrochemical reduction of SiO_2_. Unreacted SiO_2_ (if any) during the electrochemical reduction process can also be adsorbed inside the grain boundaries of Si-deposits. In addition, electrochemical reduction of SiO_2_ was carried out by applying the negative potential in the range of *E* = −1.15 and −1.25 V in the CaCl_2_ melt at a temperature of 855 °C. This potential is below the required reduction potential of CaCl_2_ (i.e., *E* = −1.30 V) as we can see in the CV diagram in Figure 1. Thus, the formation of Ca-Si alloy is unlikely in the Si-deposits obtained in this study. Still, obtained Si-deposits may contain contamination of Fe, Al, C, etc. originating from other possible sources such as steel flanges in the electrochemical cell, graphite counter electrode, Al_2_O_3_ crucible, etc.

Nevertheless, based on the above discussion, we can explain the reduction process as follows. When the applied potential at the graphite-WE (vs. Mo-RE) is more negative than the required reduction potential of SiO_2_, then nano-SiO_2_, adjacent to the graphite-WE in high-temperature melt receives electrons from conductive graphite, and become reduced to make Si-layer on graphite according to the following equation.

SiO_2_ +4e^−^→ Si + 2O^2^^−^ (3)

The formed thin layer of Si then plays the role of conducting medium at high temperature, where oncoming melt of nano-SiO_2_ gets necessary electrons to be reduced on newly formed Si-layer. The growth mechanism, along with the morphological evolution, can be explained in relation to the growth parameters as follows:

(a) Substrate: Initially, substrate materials (along with the size of the source materials) determine the growth orientation of Si nuclei, formed just after the reduction on the substrate. At a particular applied reduction potential, faster diffusion properties of high mobility metal substrates (e.g., Ag, Mo, etc.) or added metal particles in the solution enhance faster rate of electron transfer at the substrate/SiO_2_ interface, thus making larger sized nuclei on the substrate and promoting the lateral growth of the particles.
(b) Reduction potential: In the case of graphite- substrate, at lower reduction potentials (*low potential*), due to the lower rate of electron transfer between graphite and the SiO_2_ melt, the formation rate of nuclei is slow, making random nucleation sites of few numbers at nanoscale levels, and randomly oriented on the substrates. Further reduction of adjacent nano-SiO_2_ at the same *lower potential* promotes anisotropic and directional growth of Si nanowires or nanoparticles. On the other hand, at *high potential*, due to increased rate of electron transfer, the formation of a larger number of nuclei with increased size allows lateral growth of Si particles by agglomerating Si-islands. With the further proceeding of the reduction process, those Si-particles form larger Si-grains making a Si-layer along the growth front.
(c) Particle size of the SiO_2_: Finally, considering the higher dissolution rate, nano-SiO_2_ are more favorable to form Si-layers with larger grains in micro or polycrystalline levels at a given reduction potential due to a faster rate of electron transfer. SiO_2_ granules with larger particle sizes are suitable for the formation of Si at the nanoscale level (nanocrystals or nanowires) at the same reduction potential.

**Figure 6:**
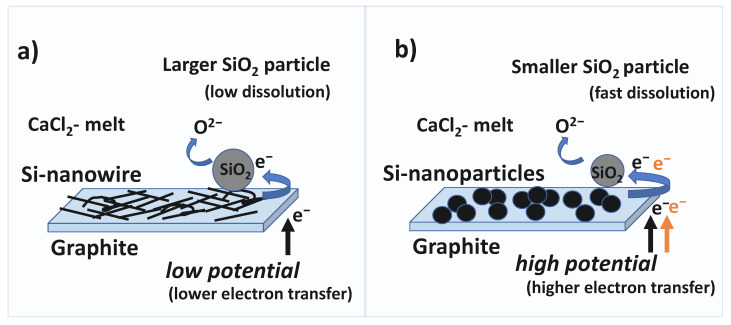
Schematic diagram of the Si growth mechanism on graphite substrates. (**a**) Favorable conditions for the formation of Si-nanowires, (**b**) favorable conditions for the formation of Si-nanoparticles.

Finally, a schematic diagram of the growth mechanism of Si-films on graphite-substrates has been shown in Figure 6, where we have considered only favorable conditions for the growth of nanowires and nano/microparticles.

As apparent from the schematic diagram, for a given substrate material, both the dissolution rate of SiO_2_ in high-temperature molten salt, and/or the transfer rate of electrons at the SiO_2_/substrate interface are crucial to determine the initial size and growth rate of nuclei formation, which later determine the morphology and microstructural properties of the final Si-deposits. Lower reduction potential and/or large-sized SiO_2_ feed materials are favorable for the formation of nanowires due to slower growth nuclei, while application of higher reduction potential and/or use of nano-SiO_2_ are favorable for the formation of laterally grown Si-nano/microparticles. In addition, application of sufficiently *low potential* alone, even with nano-SiO_2_ meets the condition for the formation of directionally grown Si-nanowires, while application of adequate *high potential* with large-size SiO_2_ particles can form nano/microparticles.

### 3.3. Optical Properties

**Figure 7:**
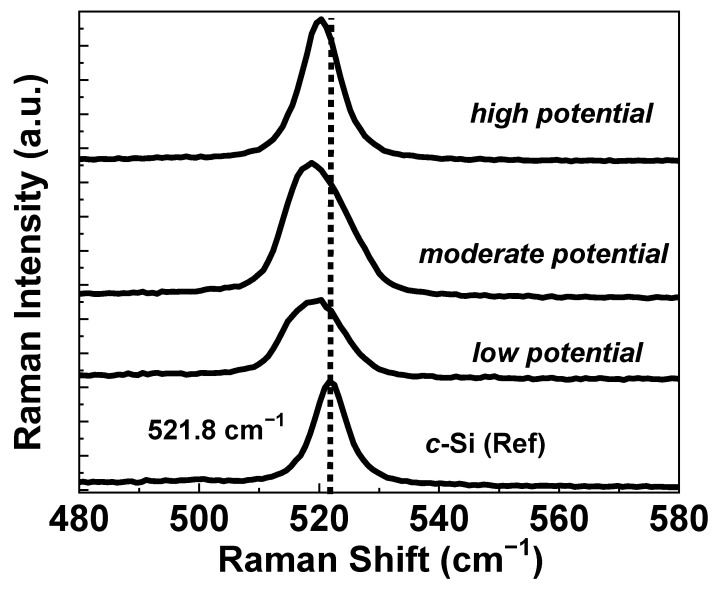
Raman spectra of electrodeposited Si-layers obtained at various reduction potentials.

To study the optical properties of the Si-layers on graphite substrates, we have performed µ-Raman spectroscopy of the Si-layers deposited with different reduction potentials as shown in Figure 7. Raman spectrum of a single crystalline silicon (*c*-Si) wafer was also shown in the same figure as a reference. In fact, Raman is a non-destructive method to identify elemental composition including phase or molecules, and also their crystallinity in the sample. As seen from the figure, *c*-Si shows a sharp and symmetric peak around 521 cm^−1^ [35], which is associated with the Raman scattering by the Si optical phonon mode. In the case of Si in the amorphous phase, the phonon peak becomes broadened and shifts to lower wavenumbers at around 480 cm^−1^ [36,37]. As seen from the figure, no such formation of the amorphous phase has been apparent in the Raman spectra of the electrodeposited films, which suggests that Si-layers obtained in this study are basically crystalline in nature (plausibly due to growth at a higher temperature of 855 °C). However, it can be seen from the figure that the Raman peak of the deposited Si-layers slightly shifted to a lower wavenumber (i.e., down shift) comparing to the *c*-Si reference peak. Shifting of the optical phonon-band to a lower wavenumber comparing to that of single-crystal Si (bulk) primarily suggests the size-related quantum confinement effect due to the formation of microcrystalline or nanocrystalline phases. In nanostructured materials, the Raman scattering selection rule is relaxed (k = 0) due to increased disorder, originating from the smaller dimension of the crystallites. It causes the first order transition of phonons to shift away from the Brillouin-zone center, causing softening and asymmetric broadening of the phonon modes with a down shift in the Raman spectra [38,39]. Broadening of the Raman peaks in the deposited Si-layers may also originate due to the size distribution of the small crystallites in the sample. It is apparent from the figure that the down-shifting of the peak becomes more prominent in the samples deposited with more lower reduction potentials.

This downshift is consistent with our observation in SEM images that samples deposited with *low potential* mainly promote the formation of Si nanowire and nanoparticles, and the diameter of the nanowire along with grain size becomes larger with an increase of the reduction potential. In addition, as seen from the figure, in general, with an increase in the applied negative potential (i.e., reduction potential), the intensity of the phonon band becomes stronger and FWHM becomes narrower. It also suggests that the crystalline quality of the samples becomes better when electrodeposition is performed with a higher negative potential vs. RE. This observation is also consistent with the XRD and SEM results as discussed before.

**Figure 8:**
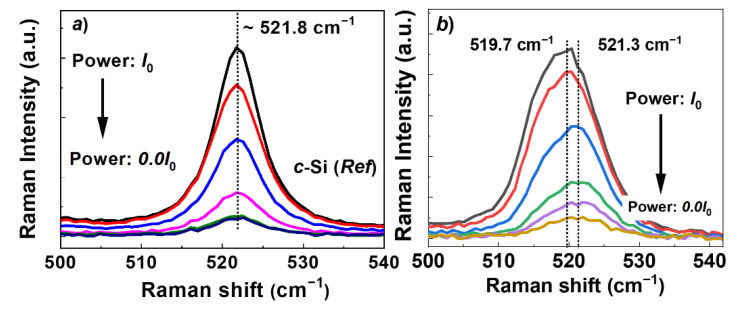
Excitation power dependence Raman spectra. (**a**) Crystalline reference silicon. (**b**) Electrodeposited Si-layer obtained with applying *low potential*.

Later, in order to confirm the size-related pure quantum confinement effect in our Si-deposits, we have performed excitation power-dependent Raman spectra. Shown in Figure 8 are the laser power-dependent Raman spectra of the *c*-Si and the Si-deposit obtained at *low potential*. As can be seen from Figure 8a, no noticeable change in the peak position of the optical phonon mode has been observed for the bulk *c*-Si sample. However, with an increase of the laser power, the Raman peak of the optical phonon mode shifts to the lower wavenumber (i.e., down shift) for the electrodeposited Si-layer (Figure 8b). The effect of laser heating on the shift of the Raman-peak in Si nanocrystals has been discussed in the literature [40,41]. A down shift of the Raman peak due to laser heating can be attributed to the expansion of the lattice of Si nanocrystals originated from the poor thermal conductivity compared to that of the bulk crystal [42]. Thus, a down shift of the Raman peak due to the pure quantum confinement effect can be obtained after subtracting the contribution from laser heating effects. Thus, from Figure 8b, for the Si-deposit obtained at *low potential* total down shift comparing to the *c*-Si can be estimated around ∆ω = 521.8 cm^−1^ − 519.7 cm^−1^ = 2.1 cm^−1^. Then, neglecting the peak shift due to the heating effect, a down shift due to the quantum confinement effect can be estimated from measurement of the Raman at lowest possible power, which yields a value of around ∆ω = 521.8 cm^−1^ − 521.3 cm^−1^ = 0.5 cm^−1^. It should be noted that tensile strain in the crystal also produces a shifting of the Raman peaks to a lower wavenumber [43]. Surface oxidation on the Si-layer may also contribute to the shift of the Raman peak. Nevertheless, a systematic downshift of the Raman peak with increasing laser power in the electrodeposited Si-layer confirms the formation of the nanostructured silicon in our study. Later, we have applied the following equation [44] to roughly estimate the average crystallite size of the Si-deposit obtained at a *low potential*, estimated from the downshift of the Raman peak due to size-related quantum confinement effect.

(4) $$d=2\pi \sqrt{\frac{B}{\Delta \omega }}$$

The term *B* = 2.24 nm^2^ cm^−1^ [45], and Δ*ω* = 521.8 cm^−1^ − 521.3 cm^−1^ = 0.5 cm^−1^ as obtained before represents the down shift of the Raman optical phonon mode compared to *c*-Si. The average crystallite size of the Si-layer using the above equation has been estimated as 13.33 nm, which is roughly similar to the calculated value of 15.15 nm obtained using Scherrer’s equation [Equation (1)] for the same sample.

**Figure 9:**
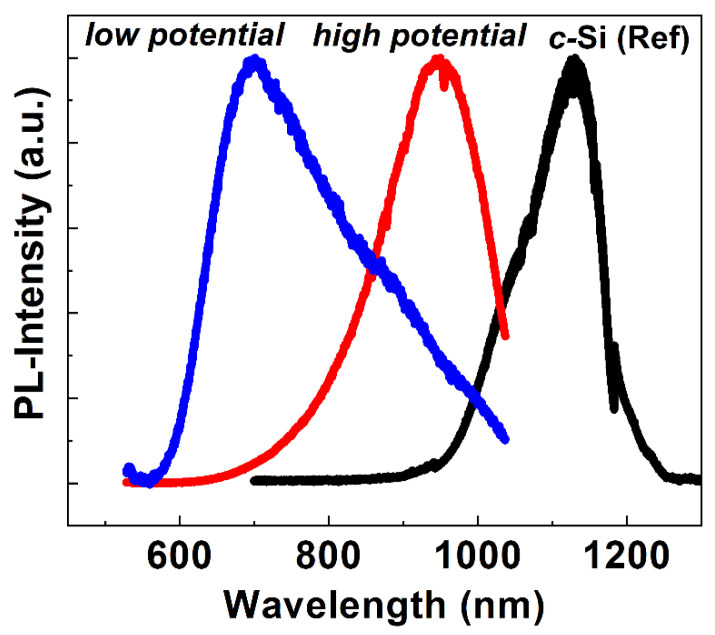
Normalized PL spectra of electrodeposited Si-layers obtained at reduction potentials set as *low* and *high potential*.

Finally, to investigate the luminescent properties of the deposited Si-layers on graphite substrates, we have performed µ-PL of the Si samples. Shown in Figure 9 are the PL spectra of the Si layers deposited with *low* and *high potential*. A PL of a single crystal silicon wafer has also been taken and plotted on the same figure. As seen from the figure, PL spectra show asymmetrically broadened features with peaks lying within the wavelength range between 650 and 950 nm, which is lower than the usual crystalline Si-peak around 1150 nm. Primarily this peak shifting suggests the widening of the bandgap of the electrodeposited Si films compared to the single crystal reference films. The widening of the bandgap can be attributed to the presence of nanowires and/or nano/microcrystalline phases in the deposited Si-films [46]. In nanocrystals, the dimensions of the semiconductors reduce from the bulk to the nanometer level and the band edges start to split into discrete states which gradually increase the optical bandgap, leading to a blue shift of PL peak position (left shift in the wavelength scale) comparing to the bulk crystal [47]. Thus, shifting of the PL peak to a higher wavelength with an increase in the reduction potentials suggests that the size of the Si crystals becomes larger, and that the crystallinity is enhanced at a higher reduction potential (as can also be seen from Raman and XRD data). It should be mentioned that PL spectra of the electrochemically obtained Si-layer could be affected by a thin, amorphous type SiO_2_ layer on the surface as discussed before. It is also worth mentioning that due to nonuniformity, the presence of mixed phases of nanowires, nanoparticles, and crystalline Si, as well as due to dendrite nature of the formed Si-layers, the peak position and intensity of micro-PL and micro-Raman may vary at different locations of a particular sample. Nevertheless, optical characterization of the Si-layers using Raman and PL along with the study of the crystal and the microstructural properties suggests that crystalline-phases, shape, size, and morphology of the electrochemically obtained Si-films on graphite substrates can be controlled by applying the appropriate reduction potential during the electrochemical reduction technique.

## 4. Conclusions

In summary, we have reported the electrochemical reduction of nano-SiO_2_ in molten CaCl_2_ at a temperature of 855 °C in a three-electrode-based electrochemical system to obtain Si-films on graphite substrates. The thickness of the obtained Si-layers was found to be ~200 µm with two hours of deposition. The electrochemical reduction mechanism has been discussed using CV, while the microstructural evolution mechanism of Si on graphite substrates was explained using SEM images. Si-films electrodeposited with lower reduction potential were mainly composed of Si nanowires at the growth font. Morphology of the deposited Si-film changed from a mixture of nanowires and nanoparticles to relatively larger microparticles with an increase of the applied reduction potential. XRD patterns and optical properties including Raman and PL also support the evolution of crystallite size of the obtained Si-layers on graphite in relation to the applied reduction potential. In addition, in general the particle size of SiO_2_ feed materials and substrate materials were also found to affect the microstructural properties of the obtained Si-films. Both the dissolution rate of SiO_2_ in high-temperature molten salt and the transfer rate of electrons at the SiO_2_/substrate interface were found to play roles in determining the morphology of the deposited Si-films. The faster diffusion rate of high mobility metal substrates, the high dissolution rate of nano-sized SiO_2_ feed materials, and faster electron transfer due to higher applied potential are suitable for forming large-sized nuclei at the initial stage of growth, which eventually promotes lateral growth of the Si particles and produces Si-films with a larger grain size. On the other hand, Si-films obtained through larger-sized SiO_2_ and/or with sufficiently lower reduction potential are composed of mainly nanowires or nanoparticles, mainly due to lower dissolution rate of larger-sized SiO_2_ and a lower rate of electron transfer at the substrate/SiO_2_ interface. Thus, selecting suitable substrate materials and controlling the size of the feed SiO_2_ particles and the applied reduction potentials during the electrochemical reduction process is crucial to obtain Si-films with desired morphology and dimensions, suitable for a particular application. Finally, the optical properties of the Si-deposits were discussed using Raman and PL spectroscopy. Particularly, the size-related pure quantum confinement effect in the Raman spectrum of the Si-deposit was manifested through a down shift of the Si optical phono mode compared to that of the bulk *c*-Si. However, in the PL spectra, a widening of the band edge in the Si-deposits confirmed the nanoscale properties of the obtained Si layers.

## Data Availability

Data is contained within the article and Supplementary Materials.

## Supplementary Materials

The following are available online at https://www.mdpi.com/article/10.3390/nano12030363/s1, Figure S1: (a) Cross-sectional SEM image of the Si-coated graphite rod after electrochemical reduction of nano SiO_2_ at *low potential* and washed with HCL, (b) SEM image of the electrodeposited Si-layer of the similar sample, with an optical image of which has been shown at the inset of (b).
