# Supplementary material for: Study of Structural and Optical Properties of Electrodeposited Silicon Films on Graphite Substrates
Source: Nanomaterials (Basel). 2022 Jan 24;12(3):363. doi: 10.3390/nano12030363 (PMC8840187; doi:10.3390/nano12030363)
Supplement: Supplementary file 1 [file nanomaterials-12-00363-s001.zip › nanomaterials-1508625-supplementary.pdf]

Supporting Information

Study of Structural and Optical Properties of Electrodeposited Silicon Films on Graphite Substrates

Muhammad Monirul Islam [1,2,*], Hajer Said [3], Ahmed Hichem Hamzaoui [3], Adel Mnif [3], Takeaki Sakurai [1], Naoki Fukata, [1,4] and Katsuhiro Akimoto [1]

[1] Institute of Applied Physics, University of Tsukuba, Tsukuba 305-8573, Ibaraki, Japan; sakurai@bk.tsukuba.ac.jp (T.S.); FUKATA.Naoki@nims.go.jp (N.F.); akimoto.katsuhiro.gf@u.tsukuba.ac.jp (K.A.)
[2] Alliance for Research on the Mediterranean and North Africa (ARENA), University of Tsukuba, Tsukuba 305-8573, Ibaraki, Japan
[3] Useful Materials Valorization Laboratory, National Centre for Research in Materials Science, Technological Park of Borj Cedria, B.P.73, Soliman 8027, Tunisia; hajer.said.doctorante@gmail.com (H.S.); mdmihi10@gmail.com (A.H.H.); mnifinrst@gmail.com (A.M.)
[4] International Center for Materials Nanoarchitectonics, National Institute for Materials Science, 1-1 Namiki, Tsukuba 305-0044, Ibaraki, Japan
* Correspondence: islam.monir.ke@u.tsukuba.ac.jp

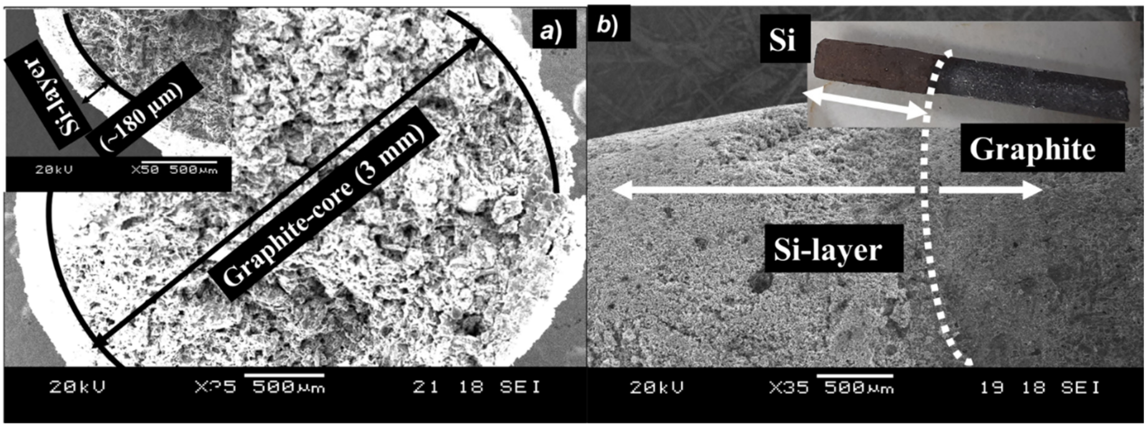

Figure S1. (**a**) Cross-sectional SEM image of the Si-coated graphite rod after electrochemical reduction of nano SiO_2 at *low potential* and washed with HCL, (**b**) SEM image of the electrodeposited Si-layer of the similar sample, an optical image of which has been shown at the inset of b).
